# Tumor Immune Microenvironments (TIMEs): Responsive Nanoplatforms for Antitumor Immunotherapy

**DOI:** 10.3389/fchem.2020.00804

**Published:** 2020-09-08

**Authors:** Xueqing Sui, Teng Jin, Tonghui Liu, Shiman Wu, Yue Wu, Zhongmin Tang, Yan Ren, Dalong Ni, Zhenwei Yao, Hua Zhang

**Affiliations:** ^1^Department of Radiology, The Affiliated Hospital of Qingdao University, Qingdao, China; ^2^Department of Radiology, Union Hospital, Tongji Medical College, Huazhong University of Science and Technology, Wuhan, China; ^3^Department of Neurology, The Affiliated Hospital of Qingdao University, Qingdao, China; ^4^Department of Radiology, Huashan Hospital, Fudan University, Shanghai, China; ^5^State Key Laboratory of High-Performance Ceramics and Superfine Microstructure, Shanghai Institute of Ceramics, Chinese Academy of Sciences, Shanghai, China; ^6^Departments of Radiology, Medical Physics, and Pharmaceutical Sciences, University of Wisconsin - Madison, Madison, WI, United States

**Keywords:** cancer, nanomaterials, enhanced immunotherapy, normalized immunotherapy, tumor immune microenvironment

## Abstract

Interest in cancer immunotherapy has rapidly risen since it offers many advantages over traditional approaches, such as high efficiency and prevention of metastasis. Efforts have primarily focused on two major strategies for regulating the body's antitumor immune response mechanisms: “enhanced immunotherapy” that aims to amplify the immune activation, and “normalized immunotherapy” that corrects the defective immune mechanism in the tumor immune microenvironments (TIMEs), which returns to the normal immune trajectory. However, due to the complexity and heterogeneity of the TIMEs, and lack of visualization research on the immunotherapy process, cancer immunotherapy has not been widely used in clinical setting. Recently, through the design and modification of nanomaterials, intelligent TIME-responsive nanoplatforms were developed from which encouraging results in many aspects of immunotherapy have been achieved. In this mini review, the status of designed nanomaterials for nanoplatform-based immune regulation of TIMEs has been emphasized, particularly with respect to the aforementioned approaches. It is envisaged that future prospects will focus on a combination of multiple immunotherapies for more efficient cancer inhibition and elimination.

## Introduction

Immunotherapy, a fast-growing tumor treatment strategy, restarts and maintains the tumor-immunity cycle to restore the body's antitumor immune response, thereby controlling and eliminating tumors (Liu et al., 2019). However, many patients have experienced minimal or no clinical benefits in response to this strategy. This has been attributed to evaded and tolerant antitumor immune responses (Gajewski et al., 2013) *via* the following mechanisms: (i) resistance to an immune attack through dominant inhibitory effects of the immune system, including suppressive pathways in infiltrated-inflamed (I-I) tumor immune microenvironments (TIMEs), brought about by programmed death-ligand 1 (PD-L1) (Gadiot et al., 2011) and tumor-associated macrophage 2 (TAM2)-type macrophages (Goswami et al., 2017), and (ii) resistance to an immune attack through immune system exclusion or ignorance in infiltrated-excluded (I-E) TIMEs in which the immune system is unable to recognize or respond to a pathogen or malignancy (Evans et al., 2016). Thus, these tumors are considered to be poorly immunogenic or “cold” (Spranger, 2016). The core of this immunosuppressive environment established in tumors is oncogenes and abnormal pathway signals, which leads to the production of potent cytokines, chemokines, and numerous immunosuppressive immune cells, finally forming the TIMEs. Broad effects of factors directly affect the quality and character of the TIMEs, such as diet (Julia et al., 2015), adiposity, the microbiome and sex, and systemic inflammatory state of an individual.

Currently, TIMEs are divided into three classes according to recent human and mouse data, helping us understand how the immune composition and immune state affect cancer cells and immunotherapy. I-E TIMEs are flooded with immune cells, but relatively lacking in cytotoxic lymphocytes (CTL) in the core of the tumor. I-E TIMEs place CTLs at the invasion boundary of tumor tissue or cause them to sink into the fibrous nest. I-I TIMEs are characterized by the high infiltration of CTLs expressed by PD-1 and cancer cells expressed by PD-1 inhibitory ligand PD-L1. Tertiary lymphoid structures (TLSs)-TIME contains a large number of lymphocytes including naive and activated conventional T cells, regulatory T cells, B cells, and protruding cells. TLS-TIMEs are usually found in the margins and stroma of aggressive tumors (Binnewies et al., 2018). Analysis of the unique classes and subclasses of TIMEs can predict and guide immunotherapeutic responsiveness and reveal new therapeutic targets (Binnewies et al., 2018). According to different treatment principles, two strategies are available, namely, normalized and enhanced tumor immunity. The former aims to reduce the suppression signal of the immune system, while the latter induces the immune system's ability to kill heterogeneous cells (Sanmamed and Chen, 2018), thereby combating tumor immune therapeutic resistance.

**Figure 1:**
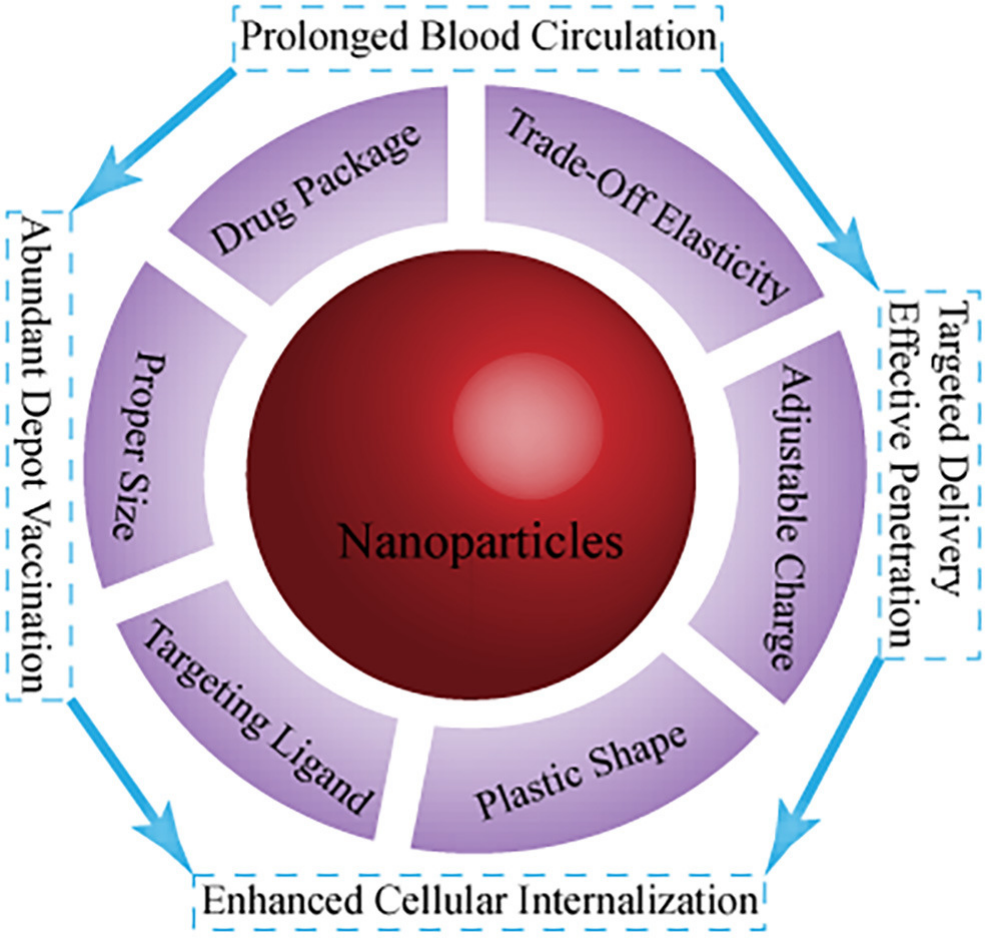
Current main strategies on the fate of immunomodulators using nanotechnology with different parameters.

Nanotechnology has been intensively investigated with respect to cancer immunotherapy. This is a key step toward creation of more effective immune responses with fewer negative implications in clinical and preclinical trials (Goldberg, 2019). Chemical modification of a nanoplatform (e.g., shape, surface charge, targeting, and responsive ability) for transport and biodistribution behavior (Figure 1) mainly focuses on (i) effective and precise delivery of immune drugs (immune antigens and cytokines, adjuvant) to targeted sites, and controlled drug release (Fan and Moon, 2015; Wang et al., 2020); (ii) optimization of the immune response to nano-tumor vaccines, enabling a variety of immune mechanisms to specifically attack and destroy cancer cells (Fu et al., 2018); (iii) regulation of immunosuppressive components of tumor immunity in the tumor microenvironment to normalize cancer immunotherapy for restoration of the lost antitumor immunity (Gao et al., 2019); and (iv) implementation of photothermal therapy (PTT) and photodynamic therapy (PDT), among others, to activate the body's immune system to improve the number and quality of antitumor immune responses, combined with cancer immunotherapy (Sang et al., 2019). However, TIME-responsive nanomaterials for cancer immunotherapy remain poorly investigated, and thus, there is scope for future work. In this minireview, the development of TIME-responsive nanomaterials has been assessed with respect to normalized and enhanced cancer immunotherapy, and perspectives for future applications are provided.

## Enhancement of Cancer Immunotherapy

Since its inception, various types of immunotherapies have been employed to activate and increase the immune response *via* modulation of general regulatory and/or activatory mechanisms (Sanmamed and Chen, 2018), involving antigen processing, activation and expansion of naive T cells, and intensification of the effector phase of the immune response. Another approach is to use effector cells/molecules of the immune system to directly attack tumor cells, consisting of antibody therapy and its derivatives, including adoptive immune cell therapy (ACT) with genetically engineered T cells, and regulation of the phenotype of immune cells in TIMEs. With the development of drug-carrying nanotechnology, significantly more antitumor molecules such as antibodies, molecular vaccines, and cytokines can be selectively delivered to target sites for augmenting retention (Bertrand et al., 2014); this is assisted by folate-, transferrin-, mannose-, and antibody-conjugated nanomaterials (Huang et al., 2016) and is combined with the EPR effect. The discovery of new drugs such as ipilimumab has paved the way for active immunotherapy by eliminating residuals and advanced cancer with durable and long-lasting responses (Hodi et al., 2010; Baronzio et al., 2013).

The presence of TAM2 in the TIME inhibits the recruitment of effector T cells to the tumor core (Beatty et al., 2015). Additionally, IL-10 and TGF-β that are excreted by TAM2 macrophages can suppress adaptive immune responses and drive the differentiation of regulatory T cells (Tregs) (Liu et al., 2019). Utilization of biomarkers that are overexpressed on tumor-associated macrophages (TAMs) to design specific ligands, construct nanocarriers, and modify their targets to remodel the TIMEs has attracted much attention, especially with respect to the polarization from TAM2 to antitumor TAM1 macrophages (Goswami et al., 2017). Additionally, albumin nanoparticles that are modified with mannose and encapsulated with drugs such as regorafenib, target albumin-binding proteins such as secreted protein acidic and rich in cysteine (SPARC) overexpressed in tumor cells and the protumor TAM2, serving both as a delivery and therapeutic strategy. TAM2 is “reeducated” into the antitumor TAM1 by means of the interplay of the TAMs, Treg, and effector CD8^+^T cells, thereby reducing apoptosis (Zhao et al., 2018). Similarly, more multifunctional nanoprobes decorated with target markers and loaded with macrophage regulators have been utilized to remodel the TIMEs *via* reprogramming of TAMs and efficiently trigger macrophage-directed cancer immunotherapy (Ai et al., 2018; Nath et al., 2018). Interestingly, natural killer cell membranes that are carried with photosensitizer-embellished nanoparticles are used synergistically with photodynamic therapy and have been found to enhance M1-macrophage polarization, inhibiting the growth of primary and distant metastatic tumors (Deng et al., 2018).

ACT is an important part of cancer immunity. Nanoprobe-based regulation mainly focuses on pre-removal of tumor suppressor T cell recruitment factors such as TGF-β to activate the T cells (Zheng et al., 2017) and on normalization of tumor vasculature with vascular endothelial growth factor (VEGF) antibodies (Stephan et al., 2015). Additionally, nanoparticles have been found to enable *ex vivo* and *in vivo* T-cell proliferation, allowing the generation of effector T cells of high quality and quantity. Iron-dextran-derived artificial antigen-presenting cells (aAPCs) were used to selectively filter tumor-specific T cells from the naïve precursors by virtue of a magnetic force (Perica et al., 2015). Nanoprobes that were conjugated with fibronectin were utilized to activate T-cell proliferation, thereby increasing the T cell expansion rate (Perica et al., 2015). Furthermore, nanoprobes loaded with DNA were selectively connected to the T cells, resulting in expression of a defined leukemia-specific Chimeric Antigen Receptor (Smith et al., 2017).

However, the therapeutic effects of ACT are impaired by insufficient proliferation and inadequate T-cell activity in the immunosuppressive TIMEs (Mardiana et al., 2019). In contrast to immunotherapy in isolation, nanoplatform-based synergistic combination cancer immunotherapy allows for improved anticancer activity as it includes radiotherapy, chemotherapy, photothermal and photodynamic therapy, gene therapy (Sang et al., 2019), and magnetic hyperthermia therapy (Pan et al., 2020). In essence, this is also a measure for enhancement of immunity by means of exposing more tumor-associated antigens, promoting the recruitment and infiltration of more effector cells into tumor tissue, and generating long-term memory T cells to prevent tumor recurrence and metastasis. Additionally, switching non-T cell-inflamed into T-cell-inflamed TIMEs can contribute to subsequent effective immunological checkpoint blockade (ICB) therapy (Spranger, 2016).

## Normalization of Cancer Immunotherapy

Compared with enhancement of cancer immunotherapy, normalized way harnesses the identification and correction of immune response deficiencies during tumor progression to further selectively restore natural antitumor immune capacity (Sanmamed and Chen, 2018). It is mainly comprised of ICB therapy, which has been widely recognized and used in clinical trials as it exhibits fewer side effects.

The B7-H1/programmed cell death-1 (PD-1) inhibitory pathway leads to the suppression of immune responses (Zou et al., 2016; Ribas and Wolchok, 2018). It has been approved for commercial use by the U.S. Food and Drug Administration (FDA) and used in clinical cancer immunotherapy (Liu et al., 2019). Accumulation and retention of checkpoint inhibitors that are encapsulated in nanoprobes within the tumor allows for enhanced efficacy (Meir et al., 2017). Furthermore, the αPD-L1 antibody combined with gold nanoparticles as the targeted marker has been harnessed to predict the benefits of anti-PD-1/PD-L1 immunotherapy through image-guided accumulation. Additionally, compared to free checkpoint inhibitors, nanomaterials loaded with PD-L1 can be decorated with tumor-targeting probes to decrease the dose while reducing side effects, and be sustainably released within the sites of interest (Teo et al., 2015; Wang C. et al., 2016). Furthermore, knockdown expression of the PD-1/PD-L1 pathway also enhances the efficacy of ICB (Shi et al., 2018). Small interfering RNA (siRNA) has been utilized to silence the PD-L1 pathway and for knockdown of PD-1 on tumor-infiltrated T cells to promote immunity against cancer and inhibit progression and metastasis of tumors (Borkner et al., 2010; Iwamura et al., 2012; Wang D. et al., 2016). Moreover, clinical trials indicate that anti-PD-1/PD-L1 combined with cytotoxic T-lymphocyte-associated protein 4 (CTLA-4) blockade exerts a synergistic antitumor effect in melanoma and lung cancer (Chae et al., 2018). Additionally, the study of inhibitory signaling pathways independence of PD-1/PD-L1 in the TIMEs will provide new strategies for nanotechnology to adjust immune normalization, such as for fibrinogen-like protein 1 (FGL1)/lymphocyte-activation gene 3 (LAG-3) (Wang et al., 2019), and the v-set immunoglobulin domain suppressor of T cell activation (VISTA) pathway (ElTanbouly et al., 2019; Mahoney and Freeman, 2020).

## Conclusions and Future Perspectives

**Figure 2:**
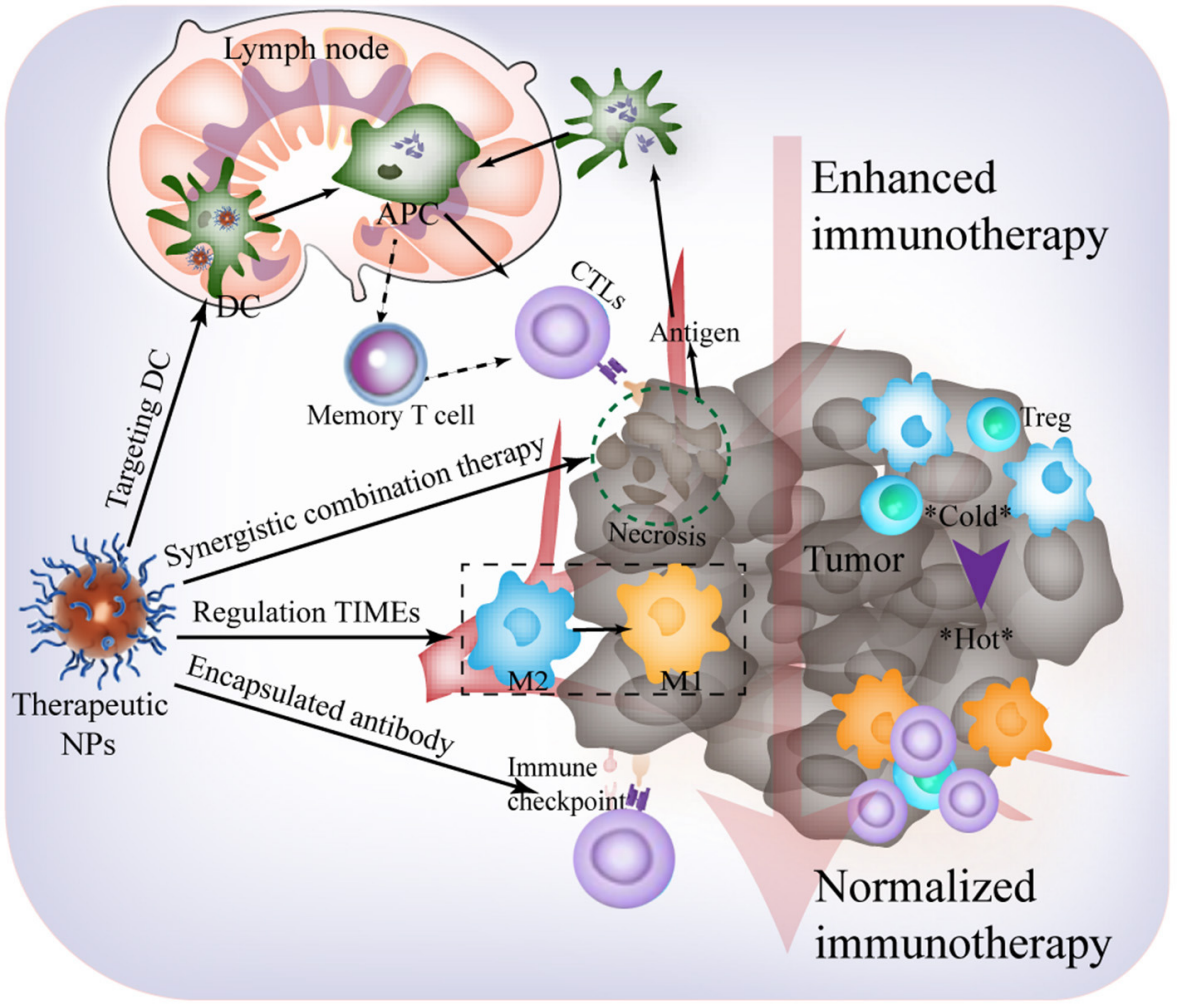
Future outlooks for effective immunological response harnessing TIME-responsive nanomaterial.

The urgent demand for effective cancer immunotherapy strategies has attracted attention in the field of biomaterials science, immunity, and molecular imaging (Liu et al., 2019). Following decades of progress, anti-TIME responses assisted by artificial nanoplatforms have been harnessed, and this is a fundamental strategy in cancer immunotherapy. However, within the development of these artificial nanomaterials, there are still many unexplored opportunities, and technical issues and scientific challenges remain to be addressed. This minireview highlights current difficulties in cancer immunotherapy and the advantages of applying nanotechnology to address immune escape and rejection. Based on previous studies (Binnewies et al., 2018), herein, we would like to provide some key points and perspectives on nanomaterial design for an effective immunological response (Figure 2). These are as follows:

1. A deeper understanding of the TIMEs can better reveal advanced biomarkers for designing nanoplatforms to exert antitumor immunotherapy. The rich immunosuppression mechanism in the tumor makes it difficult for a single treatment to standalone. The development of new tumor immune escape mechanism pathways provides more immunological checkpoint blockade targets, such as FGL1/LAG-3 and VISTA. The controlled release and multidirectional carrying characteristics of targeted nanoplatforms can comprehensively inhibit multiple immune pathways, making ICB more effective. In addition, the I-E TIMEs result in poor therapeutic effect and require warming prior to combination with other therapies, providing new ideas for effective treatment.
2. Future prospects may involve rational combination of immunotherapies with other treatments for more efficient cancer inhibition and elimination. Generally, the immune system of the patient with cancer is normal, and the focus is on the mode of usage of the composite and intelligent nanomaterials to better tune the body's immune defense to eliminate the tumor. Hence, during the design of a nanomaterial, the combination of multiple treatment methods should be considered.
3. The enhanced immune strategy frequently leads to immune-related adverse events (irAEs). To promote basic to clinical conversion, safety of nanotechnology and side effects of immunity must be comprehensively evaluated (Sanmamed and Chen, 2018).

As a collaborative study, we believe that the use of nanotechnology to achieve higher objective response rates with fewer irAEs is a promising approach. Additionally, it is envisaged that the steady development of such nanomaterials will improve the quality of life for patients with cancer and certainly promote the transformation of cancer immunotherapy from a basic study to clinical application.

## Author Contributions

XS and TJ contributed equally this work. DN, ZY, and HZ are the corresponding authors. All the authors contribute to writing this manuscript.

## Conflict of Interest

The authors declare that the research was conducted in the absence of any commercial or financial relationships that could be construed as a potential conflict of interest. The reviewer HP declared a shared affiliation, though no other collaboration, with one of the authors ZT to the handling editor.
